# Comparative Analysis of Gut Microbiota Between Wild and Captive Guizhou Snub‐Nosed Monkey (*Rhinopithecus brelichi*)

**DOI:** 10.1002/ece3.70690

**Published:** 2024-12-10

**Authors:** Xiaolong Huang, Haibo Li, Lan Zhang, Xu Zhang, Shaochuan Cheng, Yuying Yan, Wei Yang, Bingshun Meng, Zuobo Wang, Juanjuan Zhao, Jingcheng Ran

**Affiliations:** ^1^ Guizhou Academy of Forestry Guiyang China; ^2^ Key Laboratory of National Forestry and Grassland Administration on Biodiversity Conservation in Karst Mountainous Areas of Southwestern China Guizhou Academy of Forestry Guiyang China; ^3^ Guizhou Fanjingshan Forest Ecosystem Observation and Research Station Tongren China; ^4^ Fanjingshan National Nature Reserve Administration Tongren China

**Keywords:** 16S rRNA gene sequencing, gut microbiota, *Rhinopithecus brelichi*, wild and captive

## Abstract

Maintaining a healthy status is crucial for the successful captive breeding of critically endangered *Rhinopithecus brelichi* , it is conducive to ex situ conservation of this species and rejuvenation of its population. However, changes in the feeding environment and food can affect the composition and function of the gut microbiota in *R. brelichi* , ultimately impacting its health and adaptation. Herein, 16S rRNA gene sequencing was employed to determine the gut microbiota composition and functional variations between wild and captive *R. brelichi* populations. The results showed that the captive group had higher alpha diversity than the wild group, and significant differences were observed in their beta diversity. Captive and wild *R. brelichi* showed similar microbiota at the phylum level, which mainly comprised Firmicutes, Bacteroidota, and Spirochaetota, but captivity reduced the Firmicutes/Bacteroides ratio. Differential abundance analysis revealed that the relative abundance of microbiota related to cellulose degradation, such as Prevotellaceae_UCG_001, Christensenellaceae_R_7_group, *Ruminococcus*, and *Fibrobacter*, differed significantly between the two groups. Furthermore, the potential pathogens *Acinetobacter* and *Treponema* were significantly abundant in wild and captive groups, respectively. Functional predictions demonstrated that the most significant functional pathways at the second level between captive and wild monkeys were carbohydrate, amino acid, and lipid metabolisms. The captive monkeys exhibited higher digestive capacity and endocrine regulation as well as a higher risk of infectious diseases than wild monkeys. These findings can serve as a valuable theoretical basis for promoting the healthy breeding of *R. brelichi* and as a guide for future evaluation of the health of wild and captive monkeys.

## Graphical abstract

Maintaining a healthy status is crucial for the successful captive breeding of critically endangered *Rhinopithecus brelichi* , it is conducive to ex situ conservation of this species and rejuvenation of its population. Herein, 16S rRNA gene sequencing was employed to determine the gut microbiota composition and functional variations between wild and captive *R. brelichi* populations. These findings can serve as a valuable theoretical basis for promoting the healthy breeding of *R. brelichi* and as a guide for future evaluation of the health of wild and captive monkeys.

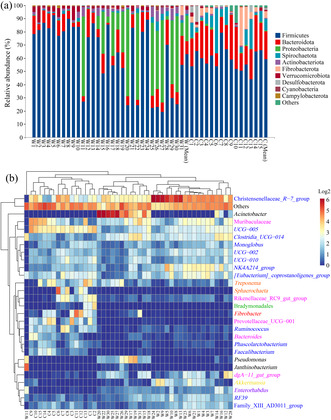

## 1 Introduction

The gut microbiota is composed of bacteria, archaea, viruses, and eukaryotic microbes, which have great potential to influence host physiology in both healthy and diseased states (Wang, Yang, et al. 2023). Over the course of evolution, a stable relationship of mutual adaptation and cooperation has developed between animals and their gut microbes, and co‐evolution has been achieved (Ley et al. 2008). The structure of the gut microbial community in animals is the result of the co‐evolution of animals and their environment. Although the gut microbial community affects the physiological functions of animals, it is highly susceptible to changes in various endogenous and exogenous factors (Bennett et al. 2016). Multiple studies have shown that the high plasticity of gut microbes makes their structure and function susceptible to changes in dietary structure, phylogenetic relationships, and the geographical environment (Zhao and Wang 2024), and phylogenetic development exerts a greater impact on the host gut microbial community than diet and geographical environment (Amato et al. 2016). However, some studies have suggested that diet plays a dominant role in determining the composition of the host gut microbiota (Hale et al. 2018; Frankel et al. 2019). A previous study reported that the environment plays a crucial role in shaping the composition of gut microbiota (Gani et al. 2024), and animals living in different environments often exhibit distinct microbial signatures (Alberdi, Bideguren, and Aizpurua 2021).

The heterogeneity of the living environment can directly influence the composition and acquisition of food resources by animals, thereby impacting the diversity of their gut microbiota (Gomez et al. 2015). The heterogeneity of primate habitats is manifested in two main ways: (1) variations between different geographical areas and (2) variations between wild and nonwild populations. A study by Zhao et al. (2018) revealed significant differences in the intestinal microbial composition of *Macaca mulatta* populations in different geographical environments. In addition, many studies have shown that the microbial composition of captive primates, such as *M. mulatta* (Jia, Chang, et al. 2022), *Macaca thibetana* (Xia et al. 2022), *Pygathrix nemaeus* (Clayton et al. 2018), *Rhinopithecus roxellanae* (Zhao et al. 2023), and *Alouatta pigra* (Nakamura et al., 2011), is significantly different from that of wild individuals, with the former showing significantly lower microbial diversity. Feeding on a single set diet may be the main cause of reduced gut microbial diversity among captive individuals (Guo et al. 2023). In addition to food‐related factors, captive individuals spend less time socializing and moving due to changes in their lifestyle (Guo et al. 2023), which may reduce the host's exposure to microbes and reduce their gut microbial diversity. However, studies have also shown the opposite trend; for instance, gorillas in captivity in zoos show higher gut microbial diversity than those living in the wild (Narat et al. 2020). The environment in which primates are held in captivity may also alter the composition of their gut microbiota; for example, the loss of the host's native microbiota due to reduced dietary fiber consumption under captive conditions (Clayton et al. 2016). It has been suggested that differences in the gut microbiome between wild and captive animals can significantly affect their overall health, particularly in terms of digestive and immune functions (Gani et al. 2024).

**FIGURE 1:**
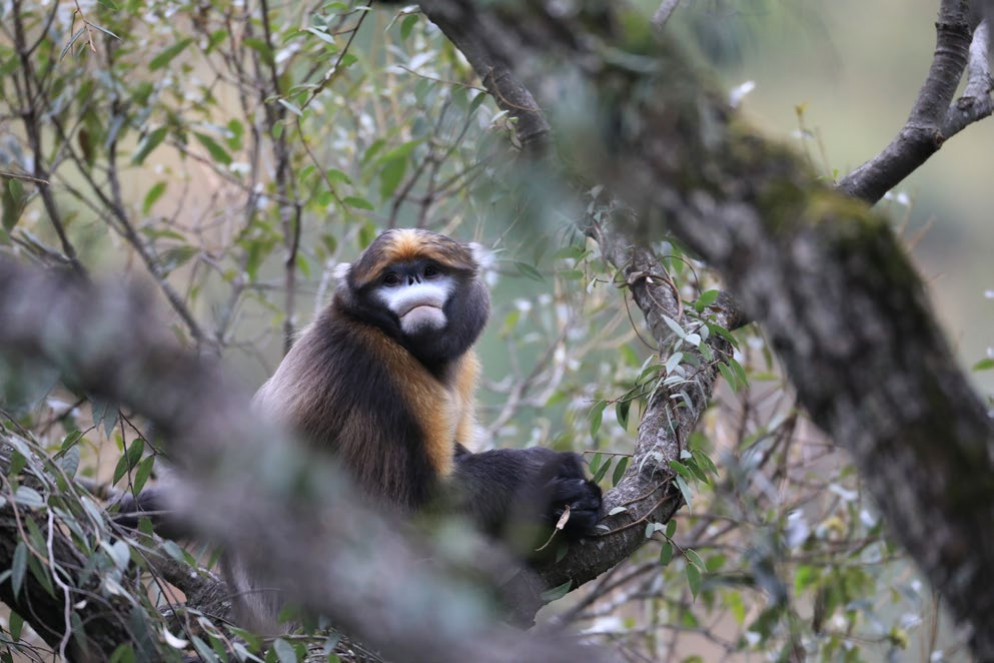
*Rhinopithecus brelichi* . A Guizhou snub‐nosed monkey is sitting in a tree. The photograph was taken by W. Yang.

The Guizhou snub‐nosed monkey ( *Rhinopithecus brelichi* ) is a primate belonging to the Cercopithecidae (Colobinae) (Figure 1) and is one of the 25 most endangered primates globally (Yang, Cui, and Niu 2023; Huang et al. 2024). It is only found in the Fanjing Mountain National Nature Reserve (FNNR) in Guizhou Province, China, and its wild population is small in number and distribution range (Jia, He, et al. 2022). Owing to habitat loss and fragmentation, the population has been isolated, making *R. brelichi* into a species with only a single continuous population, showing very low genetic diversity and slow population growth, along with a high risk of extinction. In this context, captive breeding is crucial to improve its reproductive success and help its population to recover (Yang, Cui, and Niu 2023). However, *R. brelichi* fares poorly in captivity and exhibits chronic diarrhea, poor hair coat, pale skin tone, low reproductive success, and a general failure to thrive (Hale et al. 2019). Gut microbial disorders or changes in the composition of the gut microbiome are closely related to host health. As captivity increases the contact of primates with humans, it may lead to an increase in potential pathogens in the primates' gut microbiota, thereby increasing the risk of disease (Clayton et al. 2016). Dietary differences are also an important cause of differences in gut microbes between wild and captive primates (Sun et al. 2023). *R. brelichi* is a typical leaf‐eating primate. In the wild, its diet mainly consists of a large amount of leaves (Hale et al. 2019; Zhang et al. 2024). In the captive conditions of this study, in addition to the leaves of various plants, its diet also includes a variety of fruits (grapes, bananas, dates, citrus, sweet potato, apple, pear, kiwi, mango, cantaloupe, and peach), vegetables (lettuce, romaine, carrot, pumpkin, and eggplant), sources of protein (eggs and peanuts), and coarse grains, which are not available to its counterparts in the wild. Therefore, for the ex situ conservation of these wild animals and scientific management of their captive counterparts, it is essential to understand how the lifestyle alters the gut microbial composition, revealing interactions between the gut microbiota and host.

In this study, the gut microbiota of wild and captive *R. brelichi* was evaluated using noninvasive sampling of feces and high‐throughput sequencing based on the 16S rRNA gene. Extensive studies confirmed that there are differences in the gut microbial community structure between wild and captive primates, which may be related to their diet, lifestyle, and other factors. This study aimed to provide an understanding of the composition and function of the gut microbiota in wild and captive *R. brelichi* . This study was conducted to answer the following research questions: Are there differences in gut microbial composition, diversity, and function between wild and captive *R. brelichi* ? What accounts for these differences? Are the changes in the gut microbiota of captive monkeys relevant to their health?

## 2 Materials and Methods

### 2.1 Study Area and Sample Collection

FNNR is located in the transition from Yunnan Guizhou Plateau to the hills of western Hunan and includes the main peak of Wuling Mountains (27°49′50′′ to 28°1′30′′ N, 108°45′55′′ to 108°48′30′′ E). It is located at an altitude of 500–2572 m, with an area of 419 km^2^. In terms of vegetation, it is composed of evergreen broad‐leaved, deciduous broad‐leaved, evergreen deciduous broad‐leaved mixed, and needle‐ and broad‐leaved mixed forests (Xiang et al. 2009). The FNNR is one of the areas with the highest forest biodiversity protection priority in the upper reaches of the Yangtze River. The natural vegetation in the protected area is relatively well preserved, providing a good habitat for various rare and endangered animals and plants, and this area is the only habitat for the wild population of *R. brelichi* (Wu et al. 2006).

**FIGURE 2:**
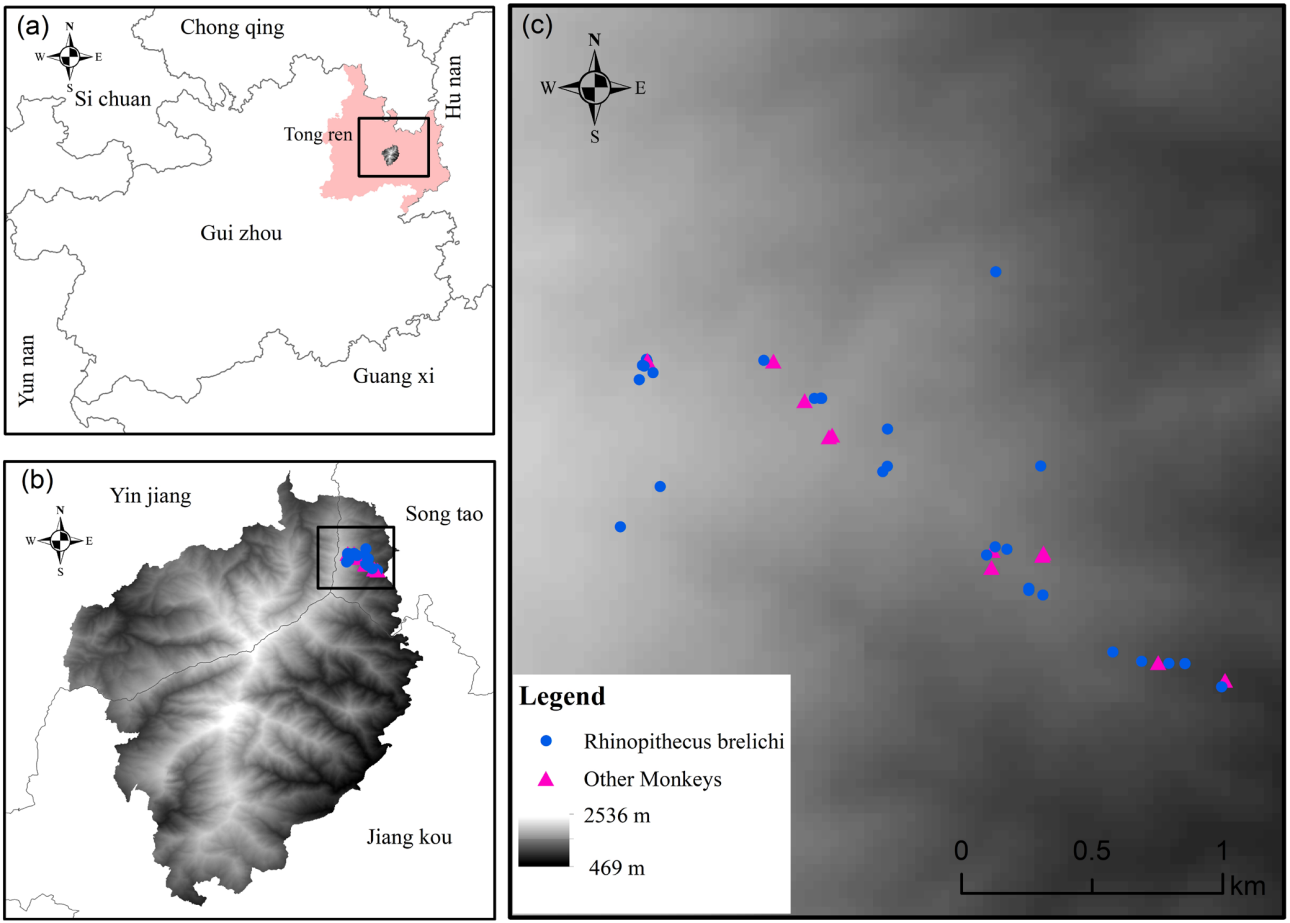
Fecal sampling points for the study of *Rhinopithecus brelichi* in Fanjing Mountain National Nature Reserve. Identification was based on the mitochondrial COI technique: 31 fecal samples belonged to *R. brelichi* and 11 to other monkey species.

From December 2021 to November 2022, fresh fecal samples were collected from all suspected *R. brelichi* in the northern part of FNNR. During this sampling, the surface soil was removed manually using disposable medical gloves. Furthermore, plant branches, leaves, and other parts attached to the samples were removed with disposable sterile tweezers and scalpels, and the middle uncontaminated part of the sample was selected and placed in 50‐mL sterile centrifuge tubes, which were then marked and packed into a sealed bag. The geographical information about the site was recorded on the sealed bags (Figure 2). To prevent DNA degradation and microbial reproduction in the fecal samples, they were immediately placed in liquid nitrogen tanks after collection and were brought back to the laboratory for storage at −80°C until use. A total of 42 fecal samples were collected in the wild, and mitochondrial CO I‐based DNA barcoding technology was used to determine whether the collected feces belonged to the *R. brelichi* , but it was still difficult to distinguish individuals. Fecal samples from captive animals were collected simultaneously using the same method. From December 2021 and November 2022, 15 samples of captive fecal samples (15 samples were collected from 5 monkeys for captive ones) were collected from the rescue station of FNNR administration. The age and sex of the five *R*. *brelichi* were 3 years old, male, 5 years old, male, 18 years old, female, 19 years old, male, and 27 years old, female.

### 2.2 DNA Extraction, Polymerase Chain Reaction (PCR) Amplification, and Sequencing

Mitochondrial CO I‐based DNA barcoding was used to identify 42 fecal samples collected in the wild, of which 31 belonged to *R. brelichi* and 11 belonged to other monkey species. Total genomic DNA samples were extracted using OMEGA DNA Kit (M5635‐02) (Omega Bio‐Tek, Norcross, GA, USA), following the manufacturer's instructions, and stored at −20°C prior to further analysis. The quantity and quality of extracted DNA were measured using a NanoDrop NC2000 spectrophotometer (Thermo Fisher Scientific, Waltham, MA, USA) and 0.8% agarose gel electrophoresis, respectively.

The V3–V4 highly variable region of the bacterial 16S rRNA gene, with a length of approximately 468 bp, was selected for PCR amplification. The primer sequences were as follows: forward primer 338F (5′‐barcode + ACTCCTACGGGAGGCAGCA‐3′, the barcode is a seven‐base oligonucleotide sequence used to distinguish different samples in the same library) and reverse primer 806R (5′‐GGACTACHVGGGTWTCTAAT‐3′) (Liu et al. 2016). PCR reactions were performed in triplicate 25 μL mixtures containing 0.25 μL of Q5 high‐fidelity DNA polymerase, 5 μL of 5× reaction buffer, 5 μL of 5× high GC buffer, 2 μL of 10 mmol L^−1^ dNTP, 1 μL of forward primer (10 μmol L^−1^), 1 μL of reverse primer (10 μmol L^−1^), and 2 μL of template DNA, with ddH_2_O added up to a final volume of 25 μL. Thermal cycling consisted of initial denaturation at 98°C for 5 min, followed by 25 cycles consisting of denaturation at 98°C for 30 s, annealing at 53°C for 30 s, and extension at 72°C for 45 s, with final extension for 5 min at 72°C (PCR instrument: ABIGeneAmp 2720, USA). PCR amplicons were purified with Vazyme VAHTS DNA Clean Beads (Vazyme, Nanjing, China) and quantified using the Quant‐iT PicoGreen dsDNA assay kit (Invitrogen, Carlsbad, CA, USA). After the individual quantification step, amplicons were pooled in equal amounts, and paired‐end 2 × 250 bp sequencing was performed using the Illumina NovaSeq platform with NovaSeq 6000 SP Reagent Kit (500 cycles) at Shanghai Personal Biotechnology Co. Ltd. (Shanghai, China).

### 2.3 Data Analysis

Microbiome bioinformatics was performed with QIIME2 (https://docs.qiime2.org/2019.4/tutorials/). Specific analysis was conducted as follows: (i) raw sequence data were demultiplexed using the demux plugin followed by primer cutting with the cutadapt plugin, invoking the primer fragment of the qiime cutadapt trim‐paired excision sequence, and discard sequence of unmatched primers (Martin 2011). (ii) Sequences were then quality filtered, denoised, and merged, chimeras were removed, low‐quality sequences, and single abundance sequences using the DADA2 plugin (Callahan et al. 2016), which obtains single nucleotide resolution based on error profiles within samples. DADA2‐denoised sequences are usually called amplicon sequence variants (ASVs). (iii) Then ASVs assigned to spike‐in sequences were filtered out and reads were counted. A standard curves (based on read counts versus spike‐in DNA copy number) for each sample were generated, and the quantitative abundance of each ASV in a sample was determined. (iv) Using 16S rRNA Greengenes database and QIIME2 classify‐sklearn algorithm (Bokulich et al. 2018): Specifically, for the feature sequence of each ASV, QIIME2 software was used with the default parameters, and species annotation was performed using a naive Bayes classifier that had been pretrained to obtain taxonomic information corresponding to each ASV.

Venn diagram was generated to visualize the shared and unique ASVs among samples or groups using R package “VennDiagram,” based on the occurrence of ASVs across groups regardless of their relative abundance. We evaluated the alpha diversity of the microbial communities using Chao1, ACE, Shannon, and Simpson indexes, as calculated with QIIME2 and visualized as box plots. Additionally, we performed Kruskal–Wallis tests to examine differences in alpha diversity between the two groups (Xi et al. 2023). Principal coordinate analysis (PCoA) and nonmetric multidimensional scaling (NMDS) were used to analyze beta diversity between the different groups to compare the intestinal microbial composition between wild and captive *R. brelichi* . Based on the Bray–Curtis similarity distance algorithm, analysis of similarities was used to test the difference between the two groups (Anderson, Ellingsen, and McArdle 2006). We used stacked bar charts to evaluate the gut microbial composition of different samples at the phylum level, and cluster heat maps to evaluate the gut microbial composition of different samples at the genus level based on the Euclidean distance clustering algorithm.

Linear discriminant analysis effect size (LEfSe) analysis was performed to identify the taxa that differed between the wild and captive groups (Segata et al. 2011). For LEfSe, Kruskal–Wallis tests were first performed among all groups of samples, followed by comparison of the selected taxa that differed between the two groups via Wilcoxon rank sum tests. Linear discriminant analysis (LDA) was used to sort the selected differences to obtain an LDA histogram (LDA score of > 3, *p* < 0.05), after which the taxonomic cladogram was obtained by mapping the differences to the classification tree with a known hierarchical structure. Clustering ASV information was compared with the sequenced microbial genome database using PICRUSt2 software to obtain the functional types and abundance of the corresponding species in the Kyoto Encyclopedia of Genes and Genomes (KEGG) database (Douglas et al. 2019) (https://www.kegg.jp/). Differences in KEGG pathways between wild and captive *R. brelichi* were analyzed using Wilcoxon rank sum tests (*p* < 0.05).

The rarefaction ASV curves, Venn diagrams, stacked column charts, LEfSe difference analysis diagrams, and KEGG function annotation diagrams were drawn using the Parsenno Genomics Cloud Platform (https://www.genescloud.cn/home). The clustering heatmaps at the genus level and PCoA and NMDS analysis charts were drawn using OmicStudio Cloud Platform (https://www.omicstudio.cn/tool). Box plots were drawn using Origin software.

## 3 Results

### 3.1 Sequence Statistics and Diversity Analysis

**FIGURE 3:**
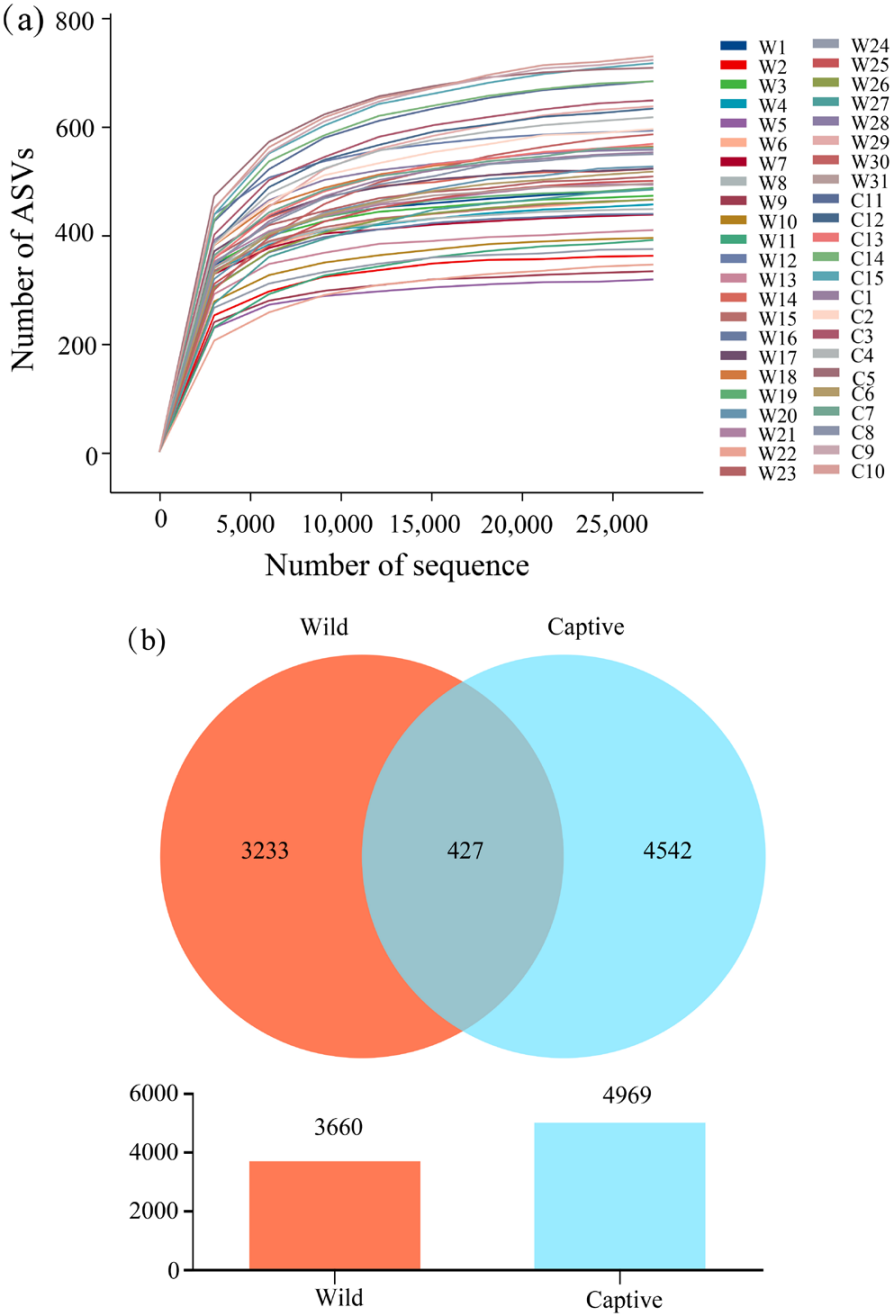
Rarefaction ASV curves (a) and Venn diagram (b) of gut microbiota of wild and captive *Rhinopithecus brelichi* . W stands for wild and C stands for captive.

A total of 4,723,744 original sequences of the target fragment, with a band size of 415.26 bp, were obtained in 46 fecal samples from wild and captive *R. brelichi*. Overall, 3,762,171 valid sequences were obtained through primer removal, splicing, mass filtration, deduplication, chimera removal, and clustering of the reads, of which 2,339,533 were from wild *R. brelichi* and 1,422,638 were from their captive counterparts. The obtained sequences with 100% similarity were clustered, and a total of 8202 ASVs were obtained, with the number of ASVs for each sample ranging from 316 to 734. The rarefaction curves based on the ASVs gradually leveled off with increasing sequencing depth, indicating that most sample information had been obtained from the sequencing data, and no more ASVs were generated with additional sequencing data (Figure 3a). Of the 8202 ASVs, 427 ASVs were shared by captive and wild *R. brelichi* , whereas 3233 and 4542 ASVs were unique to captive and wild *R. brelichi* , respectively (Figure 3b).

**FIGURE 4:**
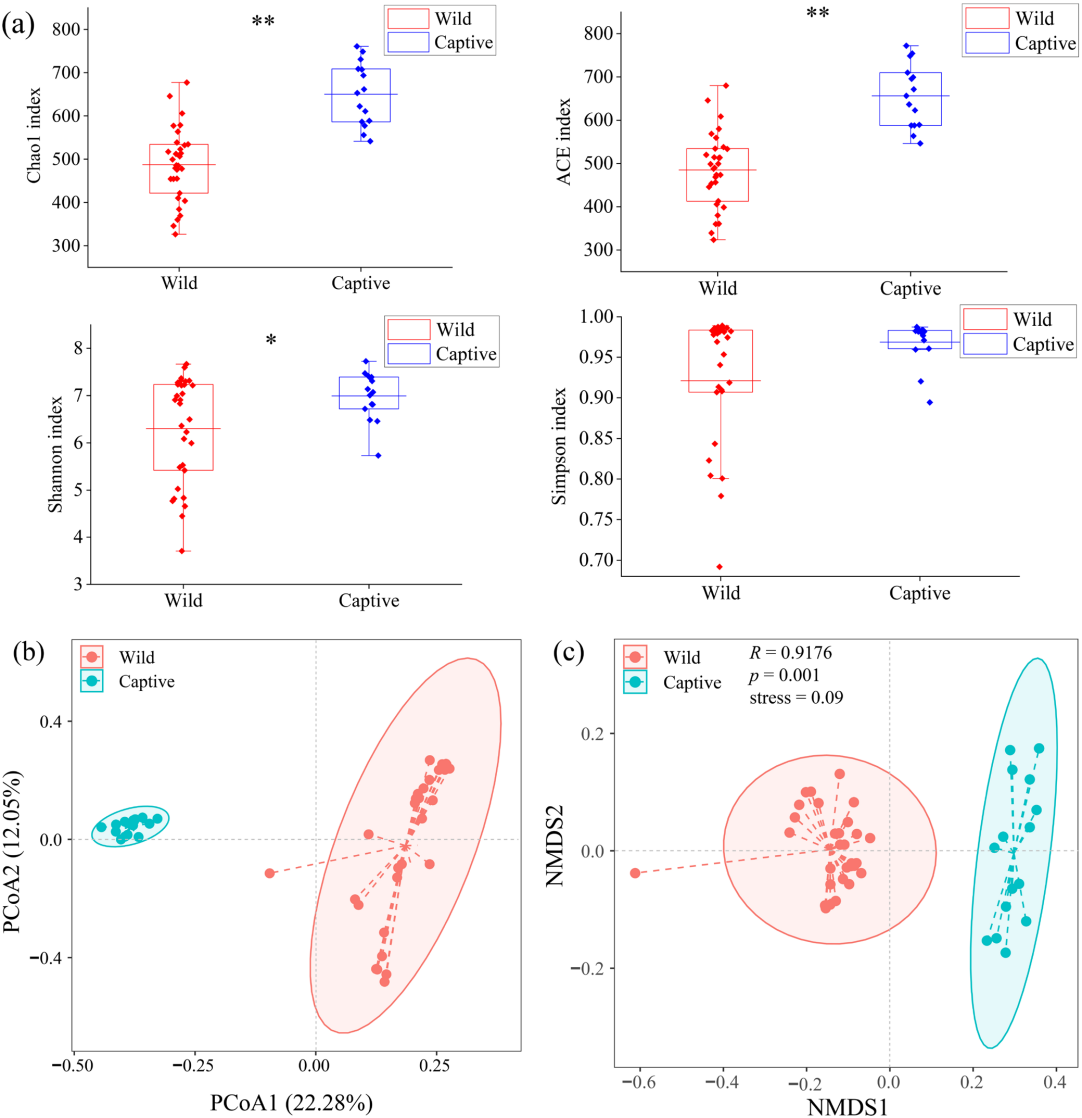
Analysis of gut microbial alpha and beta diversity in wild and captive *Rhinopithecus brelichi* . (a) The alpha diversity differences between groups as reflected in the Chao1, ACE, Shannon, and Simpson indexes were analyzed. (b) PCoA and NMDS (c) analyses based on the Bray–Curtis distance matrix. *p < 0.05, **p < 0.01.

Alpha diversity analysis showed that the average alpha diversity index of the gut microbial community of captive *R. brelichi* was higher than that of wild *R. brelichi* , and there were significant differences in Chao1, ACE, and Shannon indexes between the two groups (*p* < 0.05). This indicated that the gut microbial diversity and richness of captive *R. brelichi* were significantly higher than those of wild *R. brelichi* (Figure 4a). The beta diversity analysis based on the Bray–Curtis distance matrix showed that there were significant differences in gut microbial composition between wild and captive *R. brelichi* (*p* < 0.01). In addition, PCoA analysis revealed that wild and captive sample points were significantly separated (Figure 4b). Moreover, NMDS analysis showed that the stress value was 0.09 (less than 0.2), which could better reflect the true arrangement of the data and accurately reflect the extent of difference in the intestinal microbial composition between wild and captive *R. brelichi* (Figure 4c).

### 3.2 Gut Microbial Composition of Wild and Captive *R. brelichi*

**FIGURE 5:**
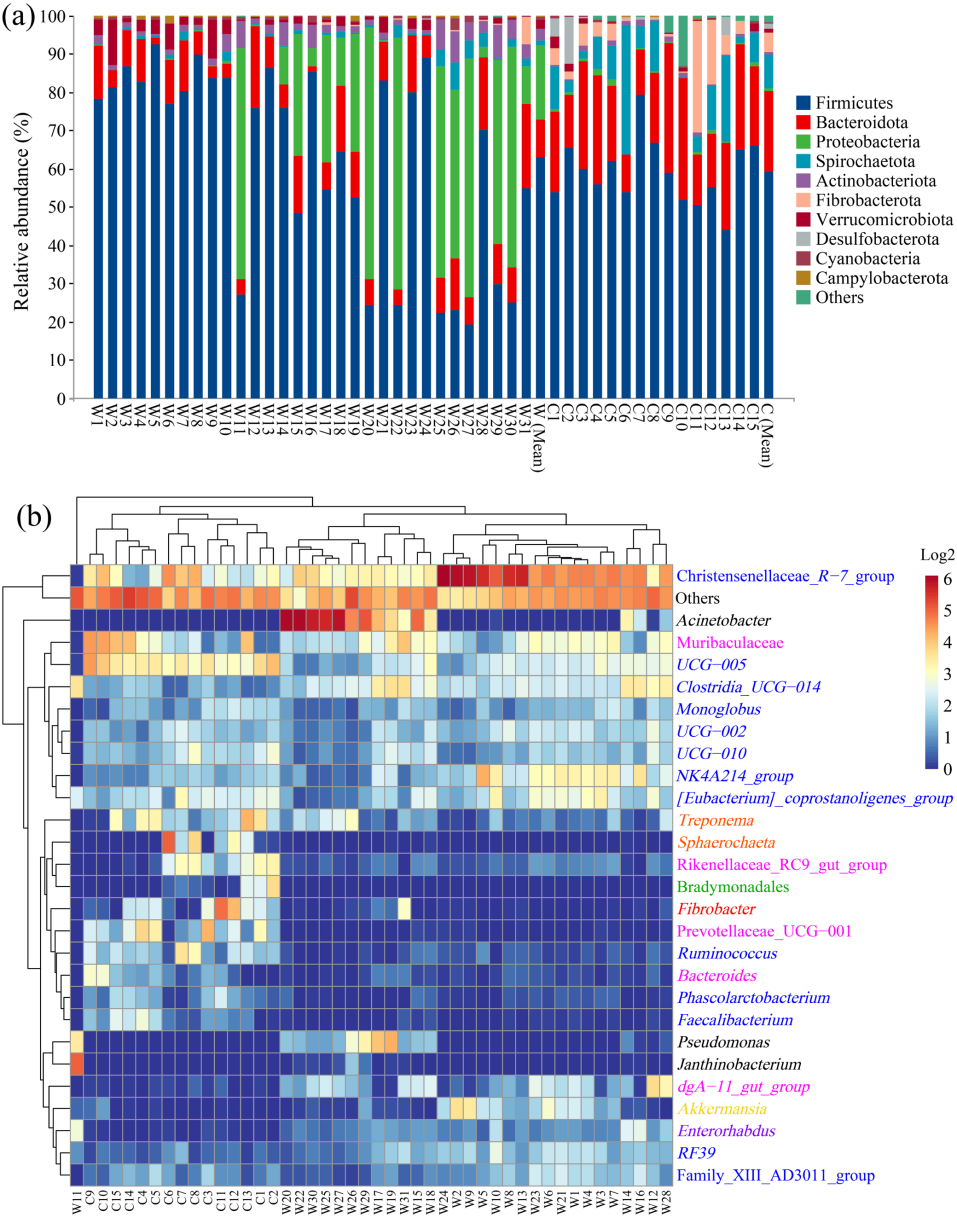
Gut microbial community composition in wild and captive *Rhinopithecus brelichi* . (a) Histogram analysis of the relative abundance of bacterial phyla. (b) Clustering heat map of bacterial genera with relative abundance of > 1%. The color scale ranges from blue (low abundance) to red (high abundance). The blue, pink, black, green, orange, red, purple, and yellow characters represent the dominant bacterial phyla of Firmicutes, Bacteroidota, Proteobacteria, Desulfobacterota, Spirochaetota, Fibrobacterota, Actinobacteriota, and Verrucomicrobiota, respectively.

We identified 8202 unique ASVs from the 46 fecal samples based on taxonomic annotation, distributed across 28 phyla, 60 classes, 126 orders, 213 families, and 497 genera. At the phylum level, Firmicutes, Bacteroidota, Verrucomicrobiota, Actinobacteriota, Spirochaetota, and Proteobacteria were the core dominant bacterial phyla in all samples, with relative abundances exceeding 1%. Campylobacterota was absent only in samples C14 and C15, and its average relative abundance was 0.23%. Meanwhile, Cyanobacteria was absent only in sample C7, and its average relative abundance was 0.45%. Moreover, Fibrobacterota was absent in samples W1, W5, W12, and C15, with an average relative abundance of 1.87%. Firmicutes, Proteobacteria, and Bacteroidota were the predominant microbes in the gut of wild *R. brelichi* , accounting for more than 92% of the total relative abundance. Firmicutes, Bacteroidota, Spirochaetota, and Fibrobacterota were the clearly dominant groups of intestinal microorganisms in captive *R. brelichi* , accounting for more than 94% of the total relative abundance. However, the relative abundance of other bacterial groups was < 5% (Figure 5a). The Firmicutes/Bacteroidota (F/B) ratio of wild *R. brelichi* was higher than that of their captive counterparts.

At the genus level, the bacteria with ≥ 1% abundance were selected for analysis and the bacteria with unclassified abundance or relative abundance of < 1% were classified as others. *UCG‐005*, *Clostridia*_UCG‐014, Christensenellaceae_*R‐7*_group, *[Eubacterium]_coprostanoligenes_group*, Muribaculaceae (unidentified genus), *NK4A214_group*, *Treponema*, *UCG‐002*, *UCG‐010*, *Monoglobus*, *RF39*, *Ruminococcus*, and *Enterorhabdus* were bacterial genera that were common to all samples. The composition of gut microbial dominant groups differed between wild and captive *R. brelichi* . In the gut microbiota of wild *R. brelichi* , the Christensenellaceae_*R‐7*_group showed the highest abundance, followed by *Acinetobacter* and *Clostridia_UCG‐014*. Meanwhile, *UCG‐005* was the most dominant genus of captive *R. brelichi* , followed by Christensenellaceae_*R‐7*_group, Muribaculaceae (unidentified genus), and *Fibrobacter*. Other dominant genera identified in captive individuals included *Treponema* and *Sphaerochaeta*, both belonging to the phylum Spirochaetota. The clustering results indicated that wild and captive individuals clustered into a single group (Figure 5b).

### 3.3 Analysis of Gut Microbial Differences Between Wild and Captive *R. brelichi*

**FIGURE 6:**
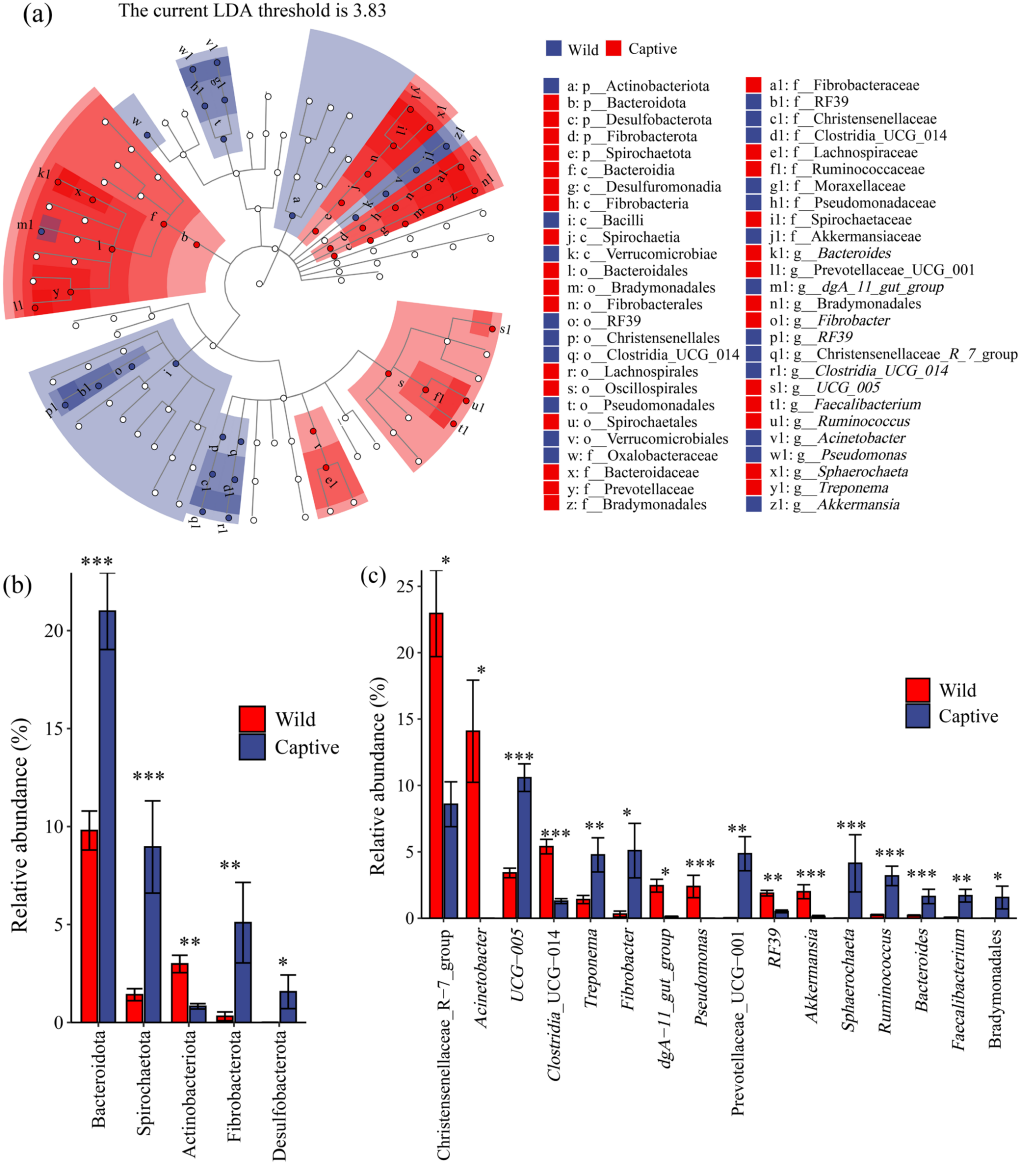
Analysis of gut microbiota difference between wild and captive. *Rhinopithecus brelichi* . (a) LEfSe analysis evolutionary branching diagram: The diagram shows phylum, class, order, family, and genus in this order from the inside to the outside. The size of the small circle indicates the relative abundance of species at the taxonomic level, with species with no significant differences marked in white and species with significant differences marked in red and blue. (b) Difference taxa at phyla level and (c) difference taxa at genera level. *p < 0.05, **p < 0.01, ***p < 0.001.

To investigate the potential differences in composition of the microbial community between wild and captive populations, LEfSe was used to analyze the gut microbes of samples from both groups (LDA score of > 3). This revealed 52 taxonomic clades with pronounced differences (Figure 6a). At the phylum level, the relative abundances of Bacteroidota, Spirochaetota, Fibrobacterota, and Desulfobacterota were significantly higher in the captive *R. brelichi* than in their wild counterparts. In contrast, the relative abundance of Actinobacteriota was significantly higher in wild *R. brelichi* than in their captive counterparts (Figure 6b). At the genus level, the relative abundances of Prevotellaceae_UCG_001, *UCG_005*, *Fibrobacter, Sphaerochaeta*, *Treponema*, *Ruminococcus*, Bradymonadales (unidentified genus), *Faecalibacterium*, and *Bacteroides* were significantly higher in the captive *R. brelichi* than in their wild counterparts. At the same time, Christensenellaceae_*R_7*_group, *Clostridia_UCG_014*, *dgA_11_gut_group*, *Pseudomonas*, *Acinetobacter*, *RF39*, and *Akkermansia* were more abundant in wild *R. brelichi* (Figure 6c).

### 3.4 Predicted Functional Differences of the Gut Microbiota Between Wild and Captive *R. brelichi*

**FIGURE 7:**
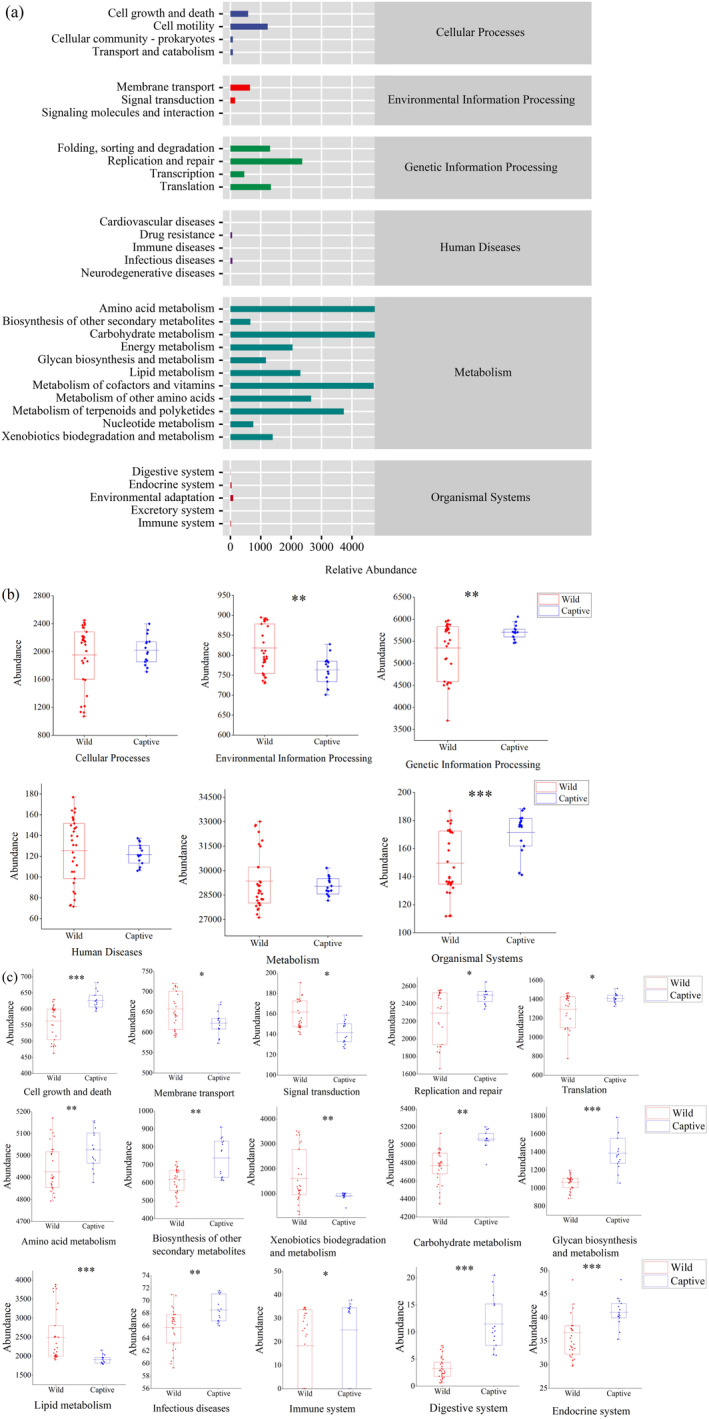
The prediction of the enrichment of KEGG pathways for all samples. (a) Annotated statistical chart of KEGG level 2 metabolic pathways in the gut microbiota of wild and captive *Rhinopithecus brelichi* . The *x*‐axis represents the relative abundance of annotations to the corresponding metabolic pathway, while the *y*‐axis corresponds to the KEGG level 2 metabolic pathways, with the level 1 category to which each metabolic pathway belongs being listed on the right. (b, c) Analysis of the differences in metabolic functions between groups at the first and second levels. *p < 0.05, **p < 0.01, ***p < 0.001.

We performed analysis of KEGG metabolic pathways in both captive and wild *R. brelichi* . In KEGG level l categories, the gut microbial genes of captive and wild *R. brelichi* were associated with six types of metabolic pathways (Figure 7a), three of which exhibited significant differences. Specifically, the enrichment of environmental information processing genes was significantly higher in wild *R. brelichi* than in captive *R. brelichi* , while the opposite pattern was observed for genes involved in genetic information processing and organismal systems (Figure 7b). In KEGG level 2 categories, the gut microbial genes of captive and wild *R. brelichi* were associated with 32 types of metabolic pathways (Figure 7a), among which 15 differed significantly (*p* < 0.05). Specifically, the captive group showed significant enrichment of pathways such as cell growth and death, replication and repair, translation, amino acid metabolism, biosynthesis of other secondary metabolites, carbohydrate metabolism, glycan biosynthesis and metabolism, infectious diseases, immune system, digestive system, and endocrine system. Meanwhile, the wild group showed significant enrichment of pathways such as membrane transport, signal transduction, xenobiotics biodegradation and metabolism, and lipid metabolism (Figure 7c).

## 4 Discussion

### 4.1 Diversity of the Gut Microbiota of Wild and Captive *R. brelichi*

The living environment of wild animals is mainly caused by environmental changes (captive breeding or semi‐release, etc.) caused by the protection of species characteristics, which affect the gut microbial composition of wild animals to a certain extent, especially endangered wild animals (Zeng 2020). Multiple studies have shown that the richness and diversity of the gut microbiota were reduced to varying degrees after captivity in *Macaca fascicularis* (Sawaswong et al. 2021), *Moschus berezovskii* (Li et al. 2017), *R. roxellanae* (Zhao et al. 2023), and *Ailuropoda melanoleuca* (Cheng et al. 2020), with some of the natural flora potentially being lost. In this study, the gut microbial richness and diversity of captive *R. brelichi* were significantly higher than those of wild *R. brelichi* , which was consistent with the results of a previous study of captive and wild *R. roxellanae* by Wang, Yang, et al. (2023), and contrary to the findings of Hale et al. (2018) on wild and captive *R. brelichi* . The captive environment increased human contact and individual interaction, which might allow a greater microbial diversity and more distinct taxa to colonize the gut tract of captive *R. brelichi*. Differences in dietary composition and quantities may also play an important role, and captive monkeys are fed adequate food at regular intervals, but wild monkeys have erratic diets because of their need to search for natural food, as well as changing weather and habitat conditions (Tang et al. 2022). As a typical leaf‐eating primate, *R. brelichi* have a relatively simple diet in the wild, mainly depend on mature leaves, while in captivity they largely depend on anthropogenic foods. In this study, *R. brelichi* in captivity eat fruits, vegetables, proteins, coarse grains, and leaves (mature leaves and young leaves), which likely cause a higher gut microbiota diversity in captive monkey than in wild. Moreover, anthropogenic food provisioning and human contact could make them susceptible to human bacteria and viruses, and this will humanize the gut microbiota composition, likely increasing the gut microbiota diversity of the captive group (Mo et al. 2023). Antibiotic use and increased sanitation may reduce microbial diversity in captivity (Hale et al. 2018; Kohl, Skopec, and Dearing 2014), while captive monkeys in our study did not ingest any drugs. Previous studies have shown that the gut microbiota of captive animals was treatment with antibiotics due to increased interactions with other animals and humans, and the accumulation of antibiotic resistance genes will reduce their adaptability and pose certain challenges to their survival (Liu et al. 2021).

### 4.2 Composition and Difference of Gut Microbiota in Wild and Captive *R. brelichi*

Wild and captive *R. brelichi* feed on different foods, and the ingestion of microorganisms via food may be one of the main sources of gut microbial colonization. In addition, these monkeys are exposed to different microorganisms through the environmental conditions in their habitat, including diet, water, soil, and social activities, which are potential sources of microbes in their gut (Diaz and Reese 2021; Sun et al. 2023). Our study revealed strong similarities in the main gut microbiota between wild and captive *R. brelichi* . The top 10 abundant phyla in abundance were distributed in both groups of fecal microbiota, among which 8 phyla were the same as the previous research result of Hale et al. (2019) on wild and captive *R. brelichi* , Desulfobacterota and Campylobacterota are absent. Moreover, Firmicutes, Bacteroidota, and Spirochaetota were the core dominant phyla of the two groups, which this has been seen in other primates such as *Rhinopithecus roxellana* (Liu et al. 2024), *Lemur catta* (Bornbusch et al. 2022), and *Trachypithecus francoisi* (Sun et al. 2023) also found similar results. These results suggest that wild and captive *R. brelichi* share a potential core gut microbiota despite their different dietary habits and living environments. This core microbiota may be essential for maintaining body function and can be retained even in the face of environmental changes (Tian et al. 2020). In contrast, the abundance of the genera Christensenellaceae_R_7_group, *Clostridia*_UCG_014, *dgA_11_gut_group, Akkermansia*, and *RF39* in wild *R. brelichi* was significantly higher than that in captive group in this study. These genera are beneficial to host digestion, metabolism, and intestinal homeostasis, which can help wild *R. brelichi* adapt to harsh conditions in the wild. For example, Christensenellaceae_R_7_group and Clostridia_UCG_014 from Firmicutes are commonly found in the intestinal tract and mucosa of the host, two genera play an important role in the degradation of cellulose and the metabolism of amino acids, peptides, lipids, and other substances, respectively (Jiang, Song, Liu, et al. 2023). As leaves rich in fibrous compounds dominate the diet of wild *R. brelichi* (Xiang et al. 2012), the significant increases in the abundance of both genera were also considered a response to changes in their diet. *Akkermansia* species are mucin degraders that live in the mucus layer of the gut and have been reported in the gut of multiple primates (Amato et al. 2015; Chen et al. 2022; Wang, Zhang, et al. 2023), which previous studies on *R. brelichi* have also demonstrated that the abundance of the genus was related to the function of its metabolic pathway (Huang et al. 2024). Hale et al. (2019) found that *Akkermansia* was more abundant in captivity because Akkermansia can utilize host mucin as its sole energy source. It can also thrive when the host is fasting or experiencing malnutrition (Van Herreweghen et al. 2018; Han et al. 2019), so the increased abundance in captivity may be due to animal malnutrition. The relatively abundant food resources in the captive environment led to a significant increase in the relative abundance of *UCG_005*, *Fibrobacter*, *Ruminococcus*, *Bacteroides*, Prevotellaceae_UCG_001, and others, which contribute to the metabolic activities of various nutrients. For example, *Fibrobacter* and *Ruminococcus* are major bacteria degrading lignocellulosic substances in the gut of leaf‐eating animals and also play an important role in carbohydrate metabolism, carbohydrate decomposition, and protein absorption (Neumann, McCormick, and Suen 2017; Neumann and Suen 2018; Chen et al. 2023). Prevotellaceae_UCG_001 belongs to the genus *Prevotella*, its enzymes can degrade cellulose and xylan, and its significant enrichment plays an important role in alleviating disordered glucose and lipid metabolism (Tang et al. 2020). The substantial increase in the abundance of these flora in the captive group may partially compensate for the significant decrease in the abundance of other cellulose‐degrading bacteria, thereby contributing to functional balance. Previous studies have also shown that *R. brelichi* has a greater capacity to use plant fibers as an energy source than *R. bieti* and *R. roxellanae* (Xi et al. 2023). This may be related to the wide distribution of cellulose‐degrading bacteria such as *UCG‐005*, *Ruminococcus*, Christensenellaceae_R‐7_group, and *Fibrobacter* in the intestine.

In addition, the abundance of *Acinetobacter* (belonging to Proteobacteria) was significantly increased in the gut of wild *R. brelichi* . This is consistent with the findings of Cabana et al. (2019), who showed that *Acinetobacter* was significantly enriched in the gut of wild *Nycticebus javanicus* but contrasts with the findings of Sun et al. (2020), who revealed significant enrichment of Proteobacteria in the gut microbiota of captive *Moschus chrysogaster* . Studies have shown that the environment, soil, and animals are the natural habitats of *Acinetobacter* for its growth and reproduction (Zhai, Wu, and Lu 2020). In humans and animals, this is associated with diseases such as septicemia, pulmonary infections, meningitis, and diarrhea, with susceptibility to infection being associated with low host resistance, resulting in disease risk (Xu et al. 2014; He et al. 2023). The abundance of this bacterial genus in the gut of wild *R. brelichi* was significantly higher than that in captivity, with such infection being suggested to occur through contaminated food and water sources, and wild *R. brelichi* also has more chances to ingest soil. There is thus a need for further research on the imbalance of *Acinetobacter* in the gut microbiota of wild *R. brelichi* , in combination with analyses of the environment and feeding. However, the potential pathogen *Treponema*, a spirochaete bacterium that can infect a wide range of hosts and tissues (Mamuad et al. 2020), was significantly increased in the gut of captive *R. brelichi* . For example, it is associated with porcine colonic spirochetosis, a diarrheal disease that can lead to reduced productivity. (Nguyen et al. 2023). A study by Zeng (2020) showed that an increase in the abundance of *Treponema* was conducive to the fermentation of cellulose and starch in *R. roxellanae*, but the role of *Treponema* in the gut of snub‐nosed monkeys remains unclear. Therefore, further studies based on the physiological characteristics of *R. brelichi* should be conducted.

### 4.3 Functional Differences of the Gut Microbiota Between Wild and Captive *R. brelichi*

Under different environmental conditions, an increase or decrease of certain gut microbiota can be an adaptation to the changing environment. In this study, the potential functions of gut microbiota in *R. brelichi* were predicted using PICRUSt2. KEGG database analysis showed that functional genes of the gut microbiota in *R. brelichi* were mainly associated with metabolic pathways such as metabolism, genetic information processing, and cellular processes, which is consistent with the results of previous studies on this species (Huang et al. 2024). KEGG functions were also influenced by food provisioning, with wild foraging monkeys showing higher functions of metabolism and environment information processing, while captive food‐fed monkeys exhibited increases in genetic information processing, cellular processes, and organismal systems, which is contrary to the results of Li et al. (2024) on Yunnan snub‐nosed monkey. At the KEGG level 2 categories, gut microbial functions of *R. brelichi* were found to be mainly enriched in carbohydrate metabolism, amino acid metabolism, and lipid metabolism, which is consistent with the findings of most researchers on the metabolic functions of gut microbiota in non‐human primates (Sun et al. 2016; Cheng et al. 2020; Wang, Yang, et al. 2023). A reduced Firmicutes/Bacteroidota ratio suggested diminished capacity for complex carbohydrate degradation in captive individuals, but the carbohydrate metabolism pathways were significantly increased in abundance in the captive group, indicating that there were other bacteria groups associated with carbohydrate metabolism that were significantly increased in abundance in the captive group. The ability to digest cellulose was decreased in captive *R. brelichi* due to changes in the diet, which in turn increased its ability to digest simple carbohydrates (Wang, Yang, et al. 2023), which is consistent with the trend of a significant increase in the abundance of carbohydrate metabolism pathways in the captive group. Meanwhile, captive *R. brelichi* exhibited enhanced digestive and endocrine regulation capabilities. Additionally, a significant increase in the enrichment of metabolic pathways related to infectious diseases was identified, suggesting a heightened risk of infectious diseases in these monkeys. Wild *R. brelichi* exhibited greater ability of the xenobiotics biodegradation and metabolism pathway, xenobiotics are manmade refractory organic pollutants that are harmful to the health of living organisms. Most of them can readily be found in various components of the environment, such as soil, sediment, and water bodies (Zhang et al. 2021). Wild *R. brelichi* live in more complex environments and are more affected by exogenous pollutants than their captive counterparts; therefore, the enhancement of this function is understandable given the greater need to maintain gut health. In addition, the enrichment of the metabolic pathway of lipid metabolism was significantly increased in the gut of wild *R. brelichi* . This may be explained by the significant increase in the bacterial genus Christensenellaceae_R‐7_group in the wild group, as a taxon mainly involved in host amino acid metabolism and lipid metabolism (Jiang, Song, Zhang, et al. 2023). Overall, captivity altered the gut microbiota of *R. brelichi* , which in turn affected their functions, but these changes may have helped the host to adapt to captivity.

## Author Contributions

**Xiaolong Huang:** conceptualization (lead), funding acquisition (lead), investigation (equal), writing – original draft (lead), writing – review and editing (equal). **Haibo Li:** methodology (equal), writing – original draft (equal), writing – review and editing (equal). **Lan Zhang:** writing – original draft (equal), writing – review and editing (equal). **Xu Zhang:** formal analysis (equal), funding acquisition (equal), visualization (equal). **Shaochuan Cheng:** formal analysis (equal), funding acquisition (equal), visualization (equal). **Yuying Yan:** data curation (equal), funding acquisition (equal). **Wei Yang:** methodology (equal). **Bingshun Meng:** resources (equal), visualization (equal). **Zuobo Wang:** formal analysis (equal), visualization (equal), writing – original draft (equal). **Juanjuan Zhao:** formal analysis (equal), visualization (equal), writing – original draft (equal). **Jingcheng Ran:** conceptualization (equal), formal analysis (equal), funding acquisition (equal), writing – review and editing (equal).

## Ethics Statement

We obtained permission to conduct this study from the FNNR Administration in Guizhou, China. This project complies with all necessary legal requirements stipulated in the Wildlife Protection Law of the People's Republic of China (NPC 2022). We have no direct interaction with *R. brelichi* that may affect their health. We collected fecal samples from forest trails and areas of high animal activity frequencies according to our previous observations.

## Conflicts of Interest

The authors declare no conflicts of interest.

## Data Availability

The dataset generated and analyzed during the current study is available in the NCBI repository: https://dataview.ncbi.nlm.nih.gov/object/PRJNA1130139?reviewer=o9alf6vgg7uubbebp8dpj7imvn.
